# Associations of CSF PDGFRβ With Aging, Blood-Brain Barrier Damage, Neuroinflammation, and Alzheimer Disease Pathologic Changes

**DOI:** 10.1212/WNL.0000000000207358

**Published:** 2023-07-04

**Authors:** Claudia Cicognola, Niklas Mattsson-Carlgren, Danielle van Westen, Henrik Zetterberg, Kaj Blennow, Sebastian Palmqvist, Khazar Ahmadi, Olof Strandberg, Erik Stomrud, Shorena Janelidze, Oskar Hansson

**Affiliations:** From the Clinical Memory Research Unit (C.C., N.M.-C., S.P., K.A., O.S., E.S., S.J., O.H.), Department of Clinical Sciences, Lund University; Memory Clinic (C.C., S.P., E.S., O.H.), Skåne University Hospital, Malmö; Department of Neurology (N.M.-C.), Skåne University Hospital, Wallenberg Center for Molecular Medicine (N.M.-C.), and Department of Clinical Sciences Lund (D.v.W.), Diagnostic Radiology, Lund University; Imaging and Function (D.v.W.), Skåne University Health Care, Lund; Department of Psychiatry and Neurochemistry (H.Z., K.B.), the Sahlgrenska Academy at the University of Gothenburg; Clinical Neurochemistry Laboratory (H.Z., K.B.), Sahlgrenska University Hospital, Mölndal, Sweden; Department of Neurodegenerative Disease (H.Z.), UCL Institute of Neurology; UK Dementia Research Institute at UCL (H.Z.), London, United Kingdom; Hong Kong Center for Neurodegenerative Diseases (H.Z.), Clear Water Bay, China; and Department of Neuropsychology (K.A.), Ruhr University Bochum, Germany.

## Abstract

**Background and Objectives:**

Injured pericytes in the neurovascular unit release platelet-derived growth factor β (PDGFRβ) into the CSF. However, it is not clear how pericyte injury contributes to Alzheimer disease (AD)–related changes and blood-brain barrier (BBB) damage. We aimed to test whether CSF PDGFRβ was associated with different AD-associated and age-associated pathologic changes leading to dementia.

**Methods:**

PDGFRβ was measured in the CSF of 771 participants with cognitively unimpaired (CU, n = 408), mild cognitive impairment (MCI, n = 175), and dementia (n = 188) from the Swedish BioFINDER-2 cohort. We then checked association with β-amyloid (Aβ)-PET and tau-PET standardized uptake value ratio, *APOE* ε4 genotype and MRI measurements of cortical thickness, white matter lesions (WMLs), and cerebral blood flow. We also analyzed the role of CSF PDGFRβ in the relationship between aging, BBB dysfunction (measured by CSF/plasma albumin ratio, QAlb), and neuroinflammation (i.e., CSF levels of YKL-40 and glial fibrillary acidic protein [GFAP], preferentially expressed in reactive astrocytes).

**Results:**

The cohort had a mean age of 67 years (CU = 62.8, MCI = 69.9, dementia = 70.4), and 50.1% were male (CU = 46.6%, MCI = 53.7%, dementia = 54.3%). Higher CSF PDGFRβ concentrations were related to higher age (*b* = 19.1, β = 0.5, 95% CI 16–22.2, *p* < 0.001), increased CSF neuroinflammatory markers of glial activation YKL-40 (*b* = 3.4, β = 0.5, 95% CI 2.8–3.9, *p* < 0.001), GFAP (*b* = 27.4, β = 0.4, 95% CI 20.9–33.9, *p* < 0.001), and worse BBB integrity measured by QAlb (*b* = 37.4, β = 0.2, 95% CI 24.9–49.9, *p* < 0.001). Age was also associated with worse BBB integrity, and this was partly mediated by PDGFRβ and neuroinflammatory markers (16%–33% of total effect). However, PDGFRβ showed no associations with *APOE* ε4 genotype, PET imaging of Aβ and tau pathology, or MRI measures of brain atrophy and WMLs (*p* > 0.05).

**Discussion:**

In summary, pericyte damage, reflected by CSF PDGFRβ, may be involved in age-related BBB disruption together with neuroinflammation, but is not related to Alzheimer-related pathologic changes.

The neurovascular unit (NVU) is an anatomic and functional complex that includes neurons, glial cells (astrocytes, oligodendrocytes, microglia), and vascular cells (endothelium, pericytes, and vascular smooth muscle cells).^1^ All these structures, and especially the vascular cells, concur in maintaining the integrity of the blood-brain barrier (BBB), a selective diffusion barrier responsible for the homeostasis of the CNS, which allows optimal synaptic and neuronal function.^1^ According to the “two-hit” hypothesis of Alzheimer disease (AD) pathogenesis, midlife cardiovascular and metabolic risk factors (e.g., hypertension and diabetes) trigger the pathologic disease cascade by causing damage to the NVU.^1,2^ It has been hypothesized that this damage to the NVU causes disruption of the BBB and reduction of cerebral blood flow (CBF, first hit), which ultimately leads to reduced β-amyloid (Aβ) clearance and formation of Aβ-containing plaques (second hit).^1^ One of the key structural and functional elements of the NVU are pericytes, which are cells that adhere to the endothelium and are involved in maintaining the BBB, while regulating CBF in the brain.^1^ The platelet-derived growth factor receptor β (PDGFRβ) is expressed in brain pericytes during cell migration and angiogenesis, and it has also been found in minor part on the surface of vascular smooth muscle cells, but not on neurons, astrocytes, endothelium, microglia, or oligodendroglia.^3^ When the BBB is damaged, PDGFRβ is released in CSF from pericytes, but not from vascular smooth muscle cells, making it a CSF marker–specific for pericyte injury.^4^ In studies where AD was diagnosed not only based on clinical symptoms but also with support of CSF biomarkers, higher levels of CSF PDGFRβ were associated with the severity of clinical symptoms and brain vascular damage.^3,5^ Furthermore, it has been proposed that CSF PDGFRβ predicts subsequent cognitive decline in *APOE* ε4 carriers.^5,6^ We also know that BBB damage increases with age and that aging is the strongest risk factor for AD dementia.^7,8^ However, it is still unclear how CSF PDGFRβ relates to aging in general and aging and key pathologic changes of AD in particular: Different studies show varying associations of CSF PDGFRβ with age and Aβ and tau CSF biomarkers.^3,5,9,10^ Large-scale clinical studies are needed to determine its association with aging, fibrillar Aβ and tau aggregates, brain atrophy, blood flow, as well as neuroinflammation and BBB integrity.

The aim of this article was to determine whether CSF PDGFRβ is indeed associated with aging and key AD pathologic changes (measured with Aβ-PET and tau-PET) and *APOE* ε4 genotype in the deeply phenotyped BioFINDER-2 cohort. Furthermore, the relationship of CSF PDGFRβ to MRI measurements of cortical thickness, white matter lesions (WML), and CBF were studied. Finally, we analyzed the role of CSF PDGFRβ in the relationship between aging, BBB dysfunction (measured by CSF/plasma albumin ratio, QAlb^11^) and neuroinflammation (i.e., CSF levels of YKL-40 and glial fibrillary acidic protein [GFAP], preferentially expressed in reactive astrocytes).

## Methods

### Standard Protocol Approvals, Registrations, and Patient Consents

All participants gave written informed consent. Ethical approval was given by the Regional Ethical Committee in Lund, Sweden.

### Study Cohort

The cohort included participants from the Swedish BioFINDER-2 study (NCT03174938). All participants were recruited at Skåne University Hospital and the Hospital of Ängelholm, Sweden. The cohort covers the full spectrum of AD, ranging from adults with intact cognition or subjective cognitive decline, mild cognitive impairment (MCI), to dementia. The main inclusion criteria, as described previously,^12^ were to be 40 years and older, being fluent in Swedish, having Mini-Mental State Examination (MMSE) scores between 27 and 30 for cognitively unimpaired (CU) participants, between 24 and 30 for MCI, and equal to or above 12 for patients with AD dementia. MCI diagnosis was established if participants performed below 1.5 SD from norms on at least 1 cognitive domain from an extensive neuropsychological battery examining verbal fluency, episodic memory, visuospatial ability, and attention/executive domains. Patients with AD dementia, vascular dementia (VaD), behavioral variant of frontotemporal dementia (bvFTD), and dementia with Lewy bodies (DLB) fulfilled the respective criteria of the *Diagnostic and Statistical Manual of Mental Disorders, Fifth Edition*.^13^ Semantic and nonfluent variants of primary progressive aphasia (svPPA, nfvPPA) were defined according to the Gorno-Tempini criteria.^14^ All patients were genotyped for *APOE*. Exclusion criteria included severe somatic disease and current alcohol/substance misuse. CSF sampling and imaging investigations were performed at the time of enrollment, in conjunction with the clinical examination and cognitive tests. The study was approved by the Regional Ethics Committee in Lund, Sweden. All participants gave written informed consent to participate.

### CSF Sampling and Analysis

CSF was collected by lumbar puncture and stored at −80°C in polypropylene tubes following the Alzheimer's Association flow chart for lumbar puncture and CSF sample processing.^15^ PDGFRβ was measured with the Human Total PDGFRβ DuoSet IC ELISA (R&D Systems Europe, Abingdon, United Kingdom) with few adaptations. In brief, the standard curve followed a 1:3 dilution, starting from 12,000 pg/mL. Capture antibody was diluted in phosphate-buffered saline (PBS). One percent MSD Blocker A buffer (cat#R93BA-4; Meso Scale Diagnostics, Rockville, MD) in PBS was used to dilute standards and as blocking buffer. Detection antibody and streptavidin were diluted in 20 mM Tris, 137 mM sodium chloride, Tween 0.05%, and 0.1% bovine serum albumin, pH 7.2–7.4. Interassay variability (coefficient of variation %) measured over 14 runs was 7.3%. For a detailed description of the protocol, see supplementary material (eMethods, links.lww.com/WNL/C795). Aβ42, Aβ40, p-tau181, YKL-40, and GFAP were measured with NeuroToolKit (Roche Diagnostics International Ltd., Mannheim, Germany). Cutoff for an Aβ-positive (Aβ+) status was calculated with the Youden index in the cohort, based on CSF Aβ42/40 (cutoff = 0.08).^12^

### Brain Imaging

Aβ-PET images were acquired on digital GE Discovery MI scanners 90–110 minutes after the injection of ∼185 megabecquerel (MBq) [18F]flutemetamol. Standardized uptake value ratio (SUVR) was calculated with pons as reference region. For the analysis, Aβ PET measures were considered both as continuous SUVR and as binarized data using a cutoff derived from mixture modeling in the BioFINDER-2 cohort (0.53 SUVR).^12^ A neocortical meta-region of interest (ROI) for Aβ-PET (prefrontal, lateral temporal, parietal, anterior cingulate, and posterior cingulate/precuneus) was calculated, as previously described.^12,16^ According to the enrollment protocol, Aβ-PET was not performed in the dementia group.

Tau-PET images were acquired on digital GE Discovery MI scanners 70–90 minutes postinjection of ∼370 MBq [18F]RO948. Tau-PET SUVR was created using the inferior cerebellar cortex as the reference region.^12^ A temporal meta-ROI for tau-PET (entorhinal cortex, inferior and middle temporal cortices, fusiform gyrus, parahippocampal cortex, and amygdala) was created, as previously described.^17^

Structural MRI was performed using a Siemens 3 T MAGNETOM Prisma scanner (Siemens Medical Solutions, Erlangen, Germany), with high-resolution T1-weighted anatomic magnetization-prepared rapid gradient echo images (1-mm isotropic voxels). T1 images underwent volumetric segmentation and parcellation using FreeSurfer (version 6.0). Cortical thickness was measured as the distance from the gray matter-white matter boundary to the perpendicular pial surface, as previously described.^18^ The AD-specific cortical thickness meta-ROI (AD signature) was measured in regions with known susceptibility to atrophy in AD (entorhinal, fusiform, inferior temporal and middle temporal regions), adjusted for cortical surface area. Automated segmentation of WML using the LST toolbox implemented in SPM8 generated a total lesion volume (in milliliters), which was then normalized for intracranial volume, as previously described.^19^ Total gray matter CBF was measured in a smaller cohort of participants in the AD continuum (CU, MCI, AD dementia, n = 392) with arterial spin labeling, see reference 20 for full method description.

### Statistics

Statistical analysis and data visualization were performed with SPSS version 26 (IBM, Armonk, NY) and R software version 4.2.3. *p* values <0.05 were considered significant. Group differences were assessed in univariate general linear models, with post hoc least significant difference tests for pairwise group comparisons. Biomarker values were log10 transformed before this analysis. Linear regression models were used to determine the associations between aging, biomarkers, and imaging measures to PDGFRβ and to test for interaction between variables. For each linear model, participants were excluded if they had 1 or more missing data in the variables included in the individual model. Mediation analysis was performed in SPSS with the PROCESS version 3.5 extension with a bootstrap method for the CIs of the mediated effect (n iterations = 5,000). Mediation effect was considered significant if the 95% CI did not include 0. Unless described otherwise, analyses were adjusted for age, sex, diagnosis, and ventricular volume. Numbers after the decimal point were rounded to the first significant figure.

### Data Availability

Anonymized data will be shared by request from any qualified investigator for the sole purpose of replicating procedures and results presented in the article and as long as data transfer is in agreement with EU legislation on the general data protection regulation.

## Results

### Study Cohort

The study cohort consisted of 771 participants diagnosed as CU patients (n = 408), patients with MCI (n = 175), or patients with dementia (n = 188) (Table 1). Disorders in the dementia group included AD (n = 124), DLB (n = 28), bvFTD (n = 13), svPPA (n = 6), nfvPPA (n = 3), and VaD (n = 14). There were, as expected, significant differences in age, *APOE* status, MMSE score, Aβ status, and Aβ-PET and tau-PET SUVR between the CU, MCI, and dementia groups (*p* < 0.001) (Table 1). Men had higher CSF levels of PDGFRβ (*p* < 0.001, eFigure 1, links.lww.com/WNL/C795). There were no differences in CSF concentrations of PDGFRβ between *APOE* ε4 carriers (1 or 2 alleles) and noncarriers (*p* > 0.05; Table 1, eFigure 2). CSF PDGFRβ concentrations did not differ between CU, MCI, and dementia groups (*p* > 0.05; eFigure 3A).

**Table 1:**
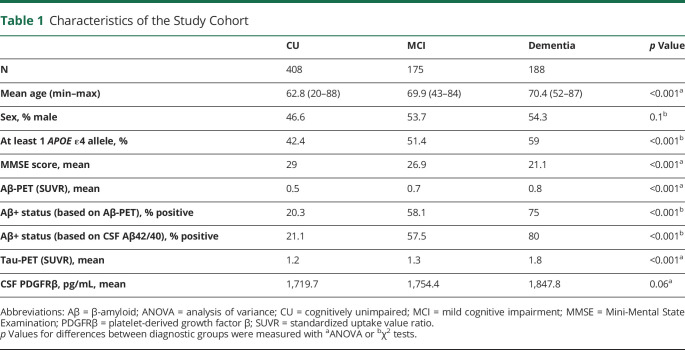
Characteristics of the Study Cohort

### Associations Between PDGFRβ and Age

**Figure 1:**
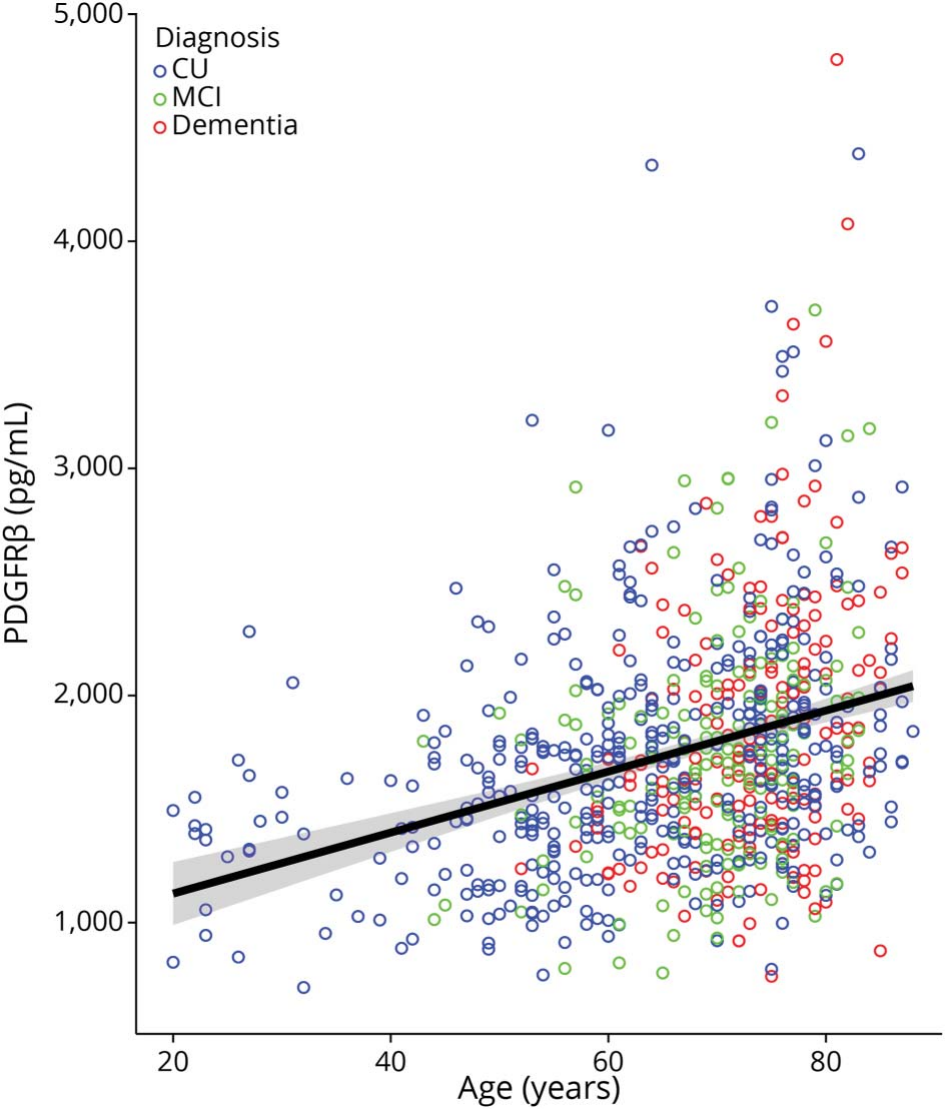
Scatter-Dot Plot Representing the Correlation Between CSF PDGFRβ and Age in the Whole Sample (n = 771) CU participants, participants with MCI, and participants with dementia shown in blue, green, and red, respectively. Regression line with 95% CIs is not adjusted for covariates. CU = cognitively unimpaired; MCI = mild cognitive impairment; PDGFRβ = platelet-derived growth factor β.

CSF PDGFRβ was overall significantly associated with age (*b* = 19.1, β = 0.5, 95% CI 16–22.2, *p* < 0.001; Figure 1). There was an interaction effect between age and diagnosis on CSF PDGFRβ (*b* = 5.3, β = 0.6, 95% CI 0.6–10, *p* = 0.03), but significant associations between age and CSF PDGFRβ survived in the diagnostic subgroups (CU: *b* = 18.7, β = 0.5, 95% CI 15–22.4, *p* < 0.001; MCI: *b* = 22.2, β = 0.4, 95% CI 13.1–31.2, *p* < 0.001; dementia: *b* = 26.5, β = 0.3, 95% CI 15.3–37.7, *p* < 0.001).

To better understand the relationship between age and PDGFRβ, we next studied whether this pericyte injury marker was associated with other age-related pathologic brain changes including key AD pathologies (Aβ and tau aggregates), small vessel disease expressed as WMLs, neuroinflammation, and BBB dysfunction.

### Associations Between PDGFRβ and AD-Related Pathologic Changes

**Figure 2:**
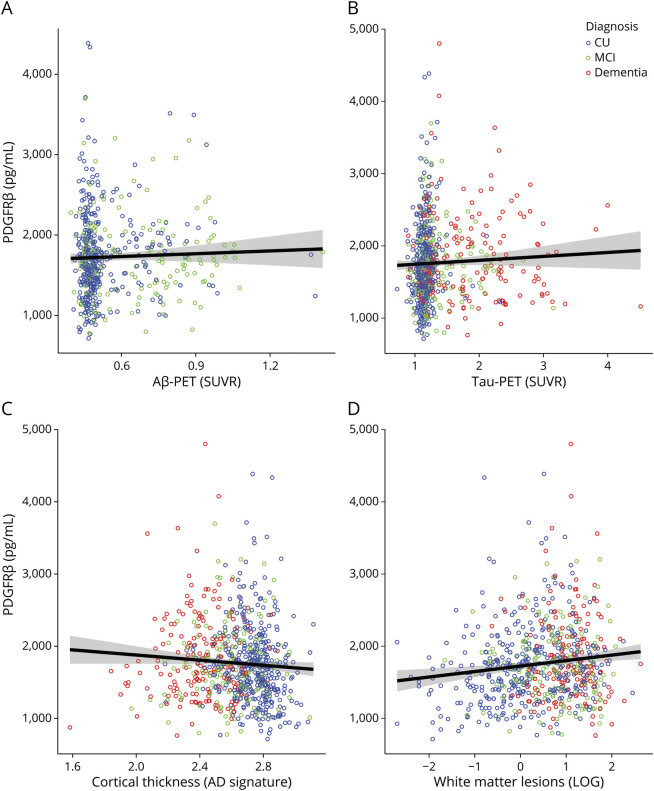
CSF PDGFRβ and AD Imaging Measures Scatter-dot plot representing the correlation between CSF PDGFRβ and Aβ-PET SUVR in the neocortical meta-ROI (A), tau-PET SUVR in the temporal meta-ROI (B), weighted cortical thickness in the AD signature meta-ROI (entorhinal, fusiform, inferior temporal, and middle temporal) (C), and volume of white matter lesions (D) in the whole sample. CU participants, participants with MCI, and participants with dementia shown in blue, green, and red, respectively. According to the study protocol, Aβ-PET was not performed in participants with dementia. Regression lines with 95% CIs are not adjusted for covariates. Aβ = β-amyloid; AD = Alzheimer disease; CU = cognitively unimpaired; MCI = mild cognitive impairment; PDGFRβ = platelet-derived growth factor β; ROI = region of interest; SUVR = standardized uptake value ratio.

CSF levels of PDGFRβ did not differ within diagnostic groups divided according to Aβ status or according to type of dementia (AD and non-AD dementias; *p* > 0.05 for all pairwise comparisons; eFigure 3B, links.lww.com/WNL/C795). Furthermore, no associations were observed between CSF PDGFRβ and Aβ-PET SUVR (n = 553, *p* > 0.05; Figure 2A) or between CSF PDGFRβ and tau-PET SUVR (n = 743, *p* > 0.05; Figure 2B). Finally, the association between age and CSF PDGFRβ was not weakened when adjusting for Aβ-PET and tau-PET (n = 544; *b* = 18.9, β = 0.5, 95% CI 15.2–22.1, *p* < 0.001). Interaction between diagnosis and Aβ-PET or tau-PET had no significant effect on CSF PDGFRβ (*p* > 0.05).

### Associations Between PDGFRβ and MRI Measures

There were no associations between CSF PDGFRβ and cortical thickness in the temporal AD signature regions (n = 749, *p* > 0.05; Figure 2C). The WML volume (n = 693, Figure 2D) and total gray matter CBF (n = 392) were not associated with the CSF levels of PDGFRβ (*p* > 0.05). The group sizes of the smaller cohort that underwent CBF analysis were consistent with those of the whole cohort (CU: n = 236 vs 408 in the whole cohort; MCI: n = 84 vs 175; dementia: n = 72 vs 188). Interaction between diagnosis and measures of cortical thickness, WML volume, or CBF had no significant effect on CSF PDGFRβ (*p* > 0.05).

### Associations Between PDGFRβ and Markers of BBB Dysfunction and Neuroinflammation

**Figure 3:**
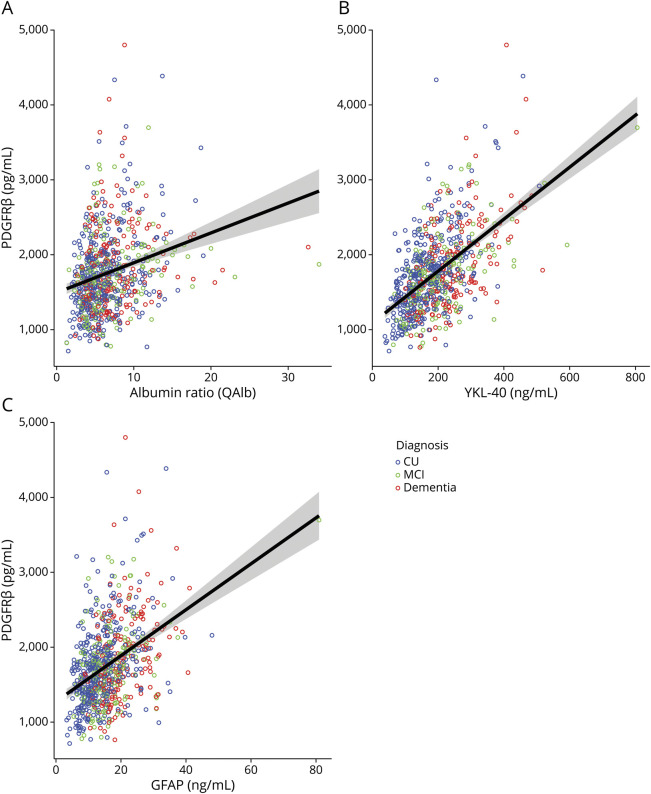
Scatter-Dot Plot Representing the Correlation Between CSF PDGFRβ and the CSF/Plasma Albumin Ratio (QAlb, A), YKL-40 (B), and GFAP (C) CU participants, participants with MCI, and participants with dementia shown in blue, green, and red, respectively. Regression lines with 95% CIs are not adjusted for covariates. CU = cognitively unimpaired; GFAP = glial fibrillary acidic protein; MCI = mild cognitive impairment; PDGFRβ = platelet-derived growth factor β.

CSF PDGFRβ was overall associated to the CSF/plasma albumin ratio (QAlb) (n = 738, *b* = 37.4, β = 0.2, 95% CI 24.9–49.9, *p* < 0.001; Figure 3A). There was a significant interaction effect between QAlb and diagnosis on the levels of CSF PDGFRβ (*b* = −25.2, β = −0.3, 95% CI −48.5 to −1.9, *p* = 0.002). Association with QAlb was not significant in the dementia subgroup (*p* > 0.05). CSF PDGFRβ levels also showed overall strong associations to the neuroinflammatory markers YKL-40 (n = 729, *b* = 3.4, β = 0.5, 95% CI 2.8–3.9, *p* < 0.001; Figure 3B) and GFAP (n = 732, *b* = 27.4, β = 0.4, 95% CI 20.9–33.9, *p* < 0.001; Figure 3C). The effect of the interaction between inflammatory markers and diagnosis on CSF PDGFRβ was not significant (*p* > 0.05).

### Analysis of the Effects of Age on BBB Dysfunction Mediated by PDGFRβ-Related Changes and Neuroinflammation

**Figure 4:**
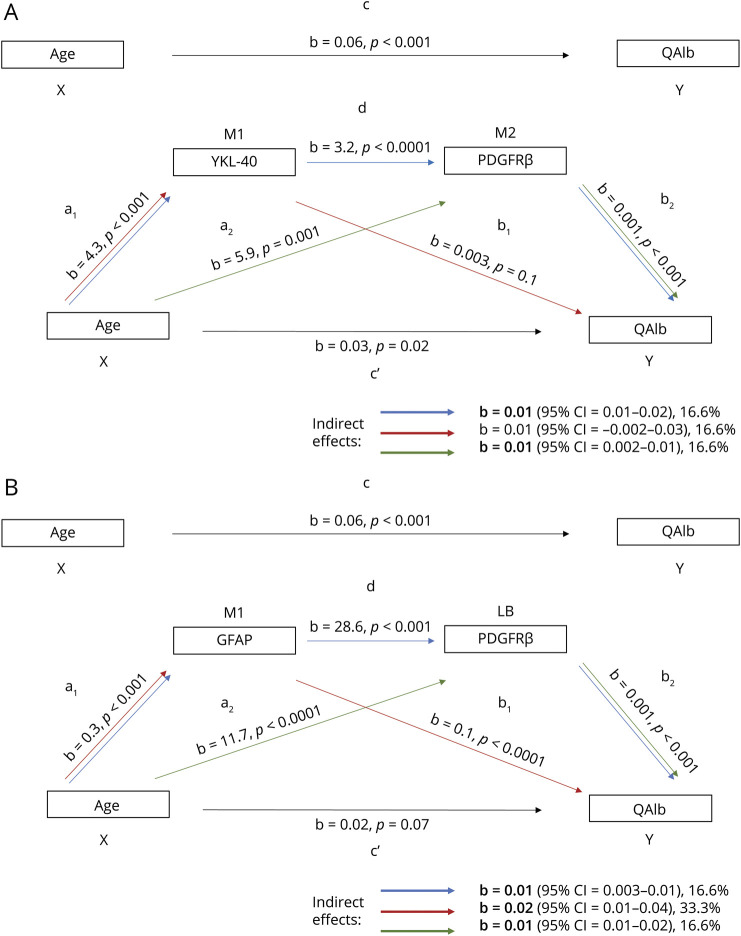
YKL-40, GFAP, and PDGFRβ as Mediators of the Effect of Age on BBB Damage Sequential mediation analysis for neuroinflammation markers (YKL-40, A; GFAP, B) and PDGFRβ (A, B) as mediators of the relationship between age (X) and CSF/plasma albumin ratio (QAlb, Y). a_1_: effect of X on M1; a_2_: effect of X on M2 adjusted for M1; b_1_: effect of M1 on Y adjusted for M2 and X; b_2_: effect of M2 on Y adjusted for M1 and X; c': direct effect of X on Y; c: total effect of X on Y; d: effect of M1 on M2 adjusted for X. Blue arrow: indirect effect for model X→M1→M2→Y; red arrow: indirect effect for model X→M1→Y; green arrow: indirect effect for model X→M2→Y. Indirect effect (coefficient indicated with *b*) was considered significant if the 95% CI did not include 0 (shown in bold). Size of the indirect effect on the total effect shown as %. GFAP = glial fibrillary acidic protein; PDGFRβ = platelet-derived growth factor β.

Because age, CSF PDGFRβ, and CSF markers reflecting neuroinflammation (YKL-40, GFAP) were associated with QAlb (eTable 1, links.lww.com/WNL/C795) and R^2^ for the models with combined effects of predictors was higher than that for individual effects (eTable 2), we performed a sequential statistical mediation analysis to determine whether neuroinflammation and pericyte damage affect the relationship between age and QAlb. We observed that CSF PDGFRβ fully mediated the effect of YKL-40 on QAlb (*b* = 0.01, β = 0.05, 95% CI 0.01–0.02, *p* < 0.05; sequential mediation shown by blue arrows in Figure 4A) because direct effect of YKL-40 on QAlb was not significant (*p* > 0.05, red arrows in Figure 4A). The indirect mediation effect of CSF PDGFRβ accounted for 16.6% of the total effect (*b* = 0.01, β = 0.02, 95% CI 0.002–0.01, *p* < 0.05; green arrows in Figure 4A). The indirect mediation effect of GFAP on QAlb accounted for 33.3% of the total effect (*b* = 0.02; β = 0.08, 95% CI 0.01–0.04, *p* < 0.05; red arrows in Figure 4B). In this model, CSF PDGFRβ showed a similar-sized (16.6%) indirect mediation effect on the total effect of age on QAlb (*b* = 0.01, β = 0.04, 95% CI 0.01–0.02, *p* < 0.05; green arrows in Figure 4B). When considering the mediators individually (not corrected for each other in the same model), they all showed a significant mediation of the effects of age on QAlb (*b* = 0.2–0.03, β = 0.1, *p* < 0.001), accounting for 33%–50% of the total effect (eFigure 4, A–C).

## Discussion

In this study, we have consistently shown that CSF PDGFRβ, a pericyte-specific marker, increases with age and is associated to BBB dysfunction (as measured by QAlb) and glial activation/neuroinflammation (CSF YKL-40 and GFAP). We also found that both age and the glial biomarkers are associated with QAlb. Interestingly, the effects of age on the BBB integrity were partially mediated by pericyte damage and neuroinflammation. CSF PDGFRβ was not related to other age-related pathologies such as AD pathologic changes, as reflected by the lack of association with *APOE* ε4 genotype or with accumulation Aβ and tau aggregates as measured with PET imaging. Levels of CSF PDGFRβ were also not related to presence of WML or changes in CBF.

Aging is associated with morphological and functional changes in BBB, and preclinical evidence indicates that age-related pericyte degeneration and reduced pericyte coverage could cause BBB breakdown, impairment of protein transcytosis, vascular damage, and alterations in blood flow (reviewed in references 7, 21). Although a previous study in living people indicated that BBB integrity loss (measured at dynamic contrast-enhanced MRI [DCE-MRI]) was age-dependent and correlated with CSF levels of PDGFRβ levels, overall investigations in clinical cohorts are few, biased by a small sample size and often reporting conflicting results.^22^ For instance, some (but not all) studies have shown correlations of PDGFRβ with age as well as with QAlb.^3,5,9,10,22,23^ Here we report that in a large cohort of well-characterized participants, older age was consistently associated with higher CSF levels of PDGFRβ and that the association was unaffected by clinical diagnosis and possible concomitant AD pathology. QAlb was also consistently associated with PDGFRβ in CU and MCI and at whole cohort level, with the exception of the dementia subgroup. Taken together, these findings provide support that age-related pericyte injury is associated with BBB dysfunction and not with AD pathology.

Aging also increases the neuroinflammatory activity in astrocytes, and astrocytic processes directly connect to the BBB in the NVU (reviewed in references 7, 21, 24). Pericytes themselves can both respond to and themselves secrete inflammatory cytokines, sustaining the local inflammation in the NVU and contributing to BBB disruption.^25-27^ Our study investigated the effect of the complex relationship between age, neuroinflammation, and pericyte damage on the integrity of the BBB in a large clinical cohort. Although we cannot prove causality through statistical mediation analysis, we lift the hypothesis that both neuroinflammation (as partly reflected by the astrocytic markers YKL-40 and GFAP) and pericyte damage mediate the effects of age on the BBB. We also propose a model where age triggers increase in neuroinflammation and pericyte damage, which are both involved in the disruption of the BBB. Furthermore, we suggest that, based on their individual and combined effects, neuroinflammation and pericyte damage interact in the disruption of the NVU.

In contrast to our findings, increases in CSF PDGFRβ concentrations have been observed in AD defined clinically or by A/T/N classification.^3,9,10,22,28^ The lack of association between CSF PDGFRβ and Aβ status and Aβ or tau biomarkers was observed previously,^3,5,9,10,22^ although 1 study showed that Aβ burden modulated the association of PDGFRβ with tau-PET.^29^ Other authors also did not find an association between PDGFRβ and small vessel disease in cerebral amyloid angiopathy participants.^9^ The existing literature has important differences from our study that need to be considered. Ours is the largest PDGFRβ clinical study to date and was conducted in a cohort characterized with not only CSF but also imaging measures. Previously, clinical groups were mostly defined based on clinical diagnosis, and the only differences in CSF PDGFRβ in groups defined by biomarkers were between A+/T+/N+ and A−/T−/N− (i.e., a difference was only seen when amyloid, tau, and neurodegeneration CSF biomarkers were pathologic, but not when only core AD biomarkers were abnormal) or within cohorts defined by A/T/N that only included preclinical AD.^3,5,9,10,22^ Most importantly, this is one of the few and the largest study using PET imaging and not only CSF biomarkers. PET imaging accurately defines the load of the core AD pathologic changes, that is, the amount and spread of insoluble Aβ and tau aggregates, which is not influenced by possible CSF dynamics that can affect biomarker concentration.^30,31^ Method-wise, some of these studies used a western blot method for detection of PDGFRβ in CSF instead of ELISA, which might have led to lower accuracy in the measurements.^5,6,22,29^ The studies where ELISA was used had a smaller sample size than ours.^3,9,10,23^ In the only study that compared the Western blot and ELISA methods in parallel, the authors suggest that the 2 techniques measure different species of PDGFRβ, which might have led to discrepancies in the results between different studies.^9^ Another possible limitation of the study is the use of QAlb to measure integrity of the BBB, which raised questions on whether this is the best method.^32^ QAlb has been shown to perform satisfactorily in this sense, especially in dementia studies^11,33^; however, more sensitive methods for detecting BBB dysfunction using MRI neuroimaging have been used in other studies,^34^ showing that BBB permeability is affected differently by AD pathology and cardiovascular risk factors. This warrants adjustment for cardiovascular risk scores in future studies.

Despite convincing evidence of the interplay between age, pericyte injury, neuroinflammation, and BBB damage, the actual extent of their role in aging and disease remains unclear. Targeted longitudinal studies in clinical cohorts and in vivo models are needed to confirm these observations and investigate the relationship between microglia, pericytes, and BBB in the aging brain.

In conclusion, we observed that the levels of CSF PDGFRβ increase with age and are associated with neuroinflammation and BBB dysfunction, but not with other age-related pathologies such as AD pathologic changes or WMLs. We also propose that pericyte damage partially mediates the disruptive effects of age on the BBB, together with neuroinflammation. Further studies are however needed to clarify the role of pericyte injury in aging, BBB dysfunction, and neurodegenerative diseases.

## Glossary

Aβ: β-amyloid
AD: Alzheimer disease
BBB: blood-brain barrier
bvFTD: behavioral variant of frontotemporal dementia
CBF: cerebral blood flow
CU: cognitively unimpaired
DLB: dementia with Lewy bodies
GFAP: glial fibrillary acidic protein
MBq: megabecquerel
MCI: mild cognitive impairment
MMSE: Mini-Mental State Examination
nfvPPA: nonfluent variant primary progressive aphasia
NVU: neurovascular unit
PBS: phosphate-buffered saline
PDGFRβ: platelet-derived growth factor β
ROI: region of interest
QAlb: CSF/plasma albumin ratio
SUVR: standardized uptake value ratio
svPPA: semantic variant primary progressive aphasia
VaD: vascular dementia
WML: white matter lesion
